# Post-Release Dispersal in Animal Translocations: Social Attraction and the “Vacuum Effect”

**DOI:** 10.1371/journal.pone.0027453

**Published:** 2011-12-14

**Authors:** Jean-Baptiste Mihoub, Alexandre Robert, Pascaline Le Gouar, François Sarrazin

**Affiliations:** 1 UMR 7204 MNHN-CNRS-UPMC, Conservation des Espèces, Restauration et Suivi des Populations, Paris, France; 2 UMR 6553 Ecobio, Université Rennes1-CNRS, Paimpont, France; Australian Wildlife Conservancy, Australia

## Abstract

Animal translocations are human-induced colonizations that can represent opportunities to contribute to the knowledge on the behavioral and demographic processes involved in the establishment of animal populations. Habitat selection behaviors, such as social cueing, have strong implications on dispersal and affect the establishment success of translocations. Using modeling simulations with a two-population network model (a translocated population and a remnant population), we investigated the consequences of four habitat selection strategies on post-translocation establishment probabilities in short- and long-lived species. Two dispersal strategies using social cues (conspecific attraction and habitat copying) were compared to random and quality-based strategies. We measured the sensitivity of local extinctions to dispersal strategies, life cycles, release frequencies, remnant population and release group sizes, the proportion of breeders and the connectivity between populations. Our results indicate that social behaviors can compromise establishment as a result of post-release dispersal, particularly in long-lived species. This behavioral mechanism, the “vacuum effect”, arises from increased emigration in populations that are small relative to neighboring populations, reducing their rate of population growth. The vacuum effect can drive small remnant populations to extinction when a translocated group is large. In addition, the magnitude of the vacuum effect varies non-linearly with connectivity. The vacuum effect represents a novel form of the behaviorally mediated Allee effect that can cause unexpected establishment failures or population extinctions in response to social cueing. Accounting for establishment probabilities as a conditional step to the persistence of populations would improve the accuracy of predicting the fates of translocated or natural (meta)populations.

## Introduction

The behavioral and demographic factors affecting the persistence of colonizing populations remain largely unknown. Indeed, many natural colonizations depend on a small number of propagules that often fail to establish a population [1]. In contrast, the widespread use and monitoring of translocations is a fruitful source of experimental data on the establishment of populations. Translocations can play a significant role in species viability through the restoration of connections among populations within a metapopulation. Translocation releases have characteristics similar to those of natural colonizations [2] and thus provide a valuable framework for investigating the demographic and behavioral processes of colonization and population establishment.

In animals, successful colonization or translocation establishment entails both the survival of individuals and their fidelity to a given area. Distinguishing between mortality and dispersal is not straightforward, and both of these processes result in local population extinction and translocation failure [3]–[5]. Despite this challenge, it is of crucial importance to identify causes of failure to adapt translocation management strategies. Dispersal and habitat selection are complex processes involving the use of environmental, individual or social information [6] and strongly influence the functional connectivity among populations within a metapopulation [7]. Information gathered by individuals through experience [8] as well as inadvertent social cues, such as the reproductive performance (i.e., habitat copying) or abundance of conspecifics (i.e., conspecific attraction), shape habitat selection in a wide range of taxa [6]. Recently, improvements in population modeling have allowed for the integration of explicit and realistic behavioral features, such as conspecific attraction, on either natural [9] or reintroduced [10] population persistence and on metapopulation dynamics [11]–[13]. However, these approaches have focused on analyses of the viability of established populations and have not investigated the impact of dispersal behaviors on the establishment of a translocated population within a metapopulation.

The initial number of individuals is generally low in both translocated and natural colonizations. Such small populations may be particularly affected by Allee effects or demographic stochasticity [14], [15], and their persistence is subject to a great deal of uncertainty [5], [16]. In both naturally occurring colonization and translocated populations, animals arrive in unfamiliar habitats in which the species may previously have been absent. The primary difference between these populations is that translocated propagules do not actively move between two habitats; they do not decide to leave their habitat of origin or to select the habitat to colonize. As a result, translocated animals frequently exhibit intense dispersal movements from the release site or maintain larger home ranges than those maintained by established or regulated natural populations (as demonstrated in mammals [17]–[19], birds [20] and reptiles [21]). In wild populations of low population size or density, habitat selection based on social cueing may initiate a behaviorally mediated Allee effect [15] if conspecific attraction leads animals to preferentially select suboptimal habitats (ecological traps [22]). This process has been shown to reduce population growth in reintroduced populations through the aggregation of animals in ecological traps after release [10]. In the present paper, we investigate the potential consequences of a behaviorally mediated Allee effect on the dynamics of translocated animal populations when (i) released animals use social cues to select their habitat and (ii) another population is present nearby. This two-population circumstance (translocated and remnant populations) may be expected to result in a strong disequilibrium in the perceived attractiveness of the habitat by the two populations, leading to a one-way dispersal pattern. Although individual fitness may be maintained, this process may directly act as a demographic Allee effect [15]. To our knowledge, the present study is the first to focus on the impact of behaviorally mediated Allee effects on the successful establishment of translocated populations.

Using a simulation-based population modeling framework, our goal is to understand the mechanisms associated with habitat selection during the establishment phase. We investigate the ways in which dispersal patterns are influenced by four habitat selection strategies based on individual and social information and how these strategies may directly affect the success of translocations under different management scenarios. Releases occur either where the species is absent from the wild (so that no conspecific population exists) or where a remnant conspecific population exists in proximity to the release area (relative to the dispersal ability of the species), i.e., when the goal of the translocation is to create a new local population within a metapopulation network. The local and global dynamics of this *translocated – remnant* population network are examined for various remnant population sizes and various degrees of connectivity between the two populations considering demographic stochasticity and stochastic dispersal events. We investigate the viability of these populations in response to different release methods and for two different life cycles.

## Methods

### Model structure

We modeled populations through an age-structured, extended Leslie population model framework [23] following a modeling approach similar to that used in Doligez et al. [24]. Using a transition matrix that includes demographic rates (survival, fecundity and dispersal) specific to different age classes, this approach allows for the description of populations in terms of abundance or age structure at time t based on their condition at t-1. This framework provides useful tools for projecting population dynamics over specified time horizons followin*g* first-order Markov chains [23]. Our model was based on a pre-breeding census with a single breeding pulse per year and a discrete annual time step. To avoid confounding effects between the factors being tested (e.g., behavioral strategies and release methods), we considered a single-sex matrix population model. This type of model is often referred to as a female life-cycle model in which i) vital rates and behaviors are assumed to be identical in males and females, ii) every female that decides to reproduce can find a mate (as females are usually considered to be the sex that limits a population growth rate) and iii) males are not explicitly described (see [23], specifically Chapter 17). Throughout the paper, population and release-group sizes are expressed in terms of the number of females (the number of mature females is regarded as a proxy for potential mating events). However, to avoid confusion, we use “female” only as a quantitative proxy for population or release group size, whereas we use the term “individual” when discussing more qualitative processes because we assume no difference between sexes with regard to behaviors such as habitat selection strategies. Importantly, considering female life cycles can, in some cases, lead to an underestimation of the risk of extinction [25] because male scarcity or a biased operational sex-ratio can affect female fitness and population dynamics [26]. However, previous theoretical studies suggest that the strength of these effects strongly depends on the mating system considered [14], [25]. In the present study, we focused on qualitative comparisons of the extinction risks involved with various behavioral and management scenarios; our simplified assumptions are not likely to affect our general conclusions in comparison with more detailed models and enable us to provide generalizable results.

**Figure 1:**
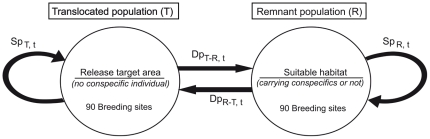
Schematic representation of the metapopulation network condired for both the *translocated* and the *remnant* populations. The two populations offer habitat of equal mean quality and carrying capacity (90 breeding sites). Habitat qualities varied however under independent local environmental stochasticity. Releases occurred in the target *translocated* population (T) only. At the beginning of the simulations, the *remnant* population (R) can either be an empty suitable habitat or an area inhabited by conspecifics. Individuals can stay in the population x at time t (staying probability Sp_x, t_) or disperse from population x to the other population y (dispersal probability Dp_x-y, t_) according to different behavioural dispersing rules.

Two local populations were depicted: the *translocated* population, where releases occurred, and the *remnant* population, which was a conspecific population distinct from the translocated population and located at another spatially distinct and suitable site (Fig. 1). Individuals were able to move from one population to the other according to behavioral dispersal rules (see the *Dispersal decisions* section). We incorporated environmental stochasticity on habitat quality, in which each population was affected independently from the other by temporal environmental variations. The two sites were assumed to have similar environmental characteristics, with an identical carrying capacity of 90 breeding sites and an equal mean habitat quality with the same variance (see below). The habitat quality of site *x* at time t (q_x,t_) was assumed to affect reproduction only and was positively autocorrelated between t and t+1:
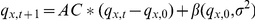
where AC is the temporal autocorrelation coefficient, β(q_x,0_, σ^2^) is a beta function of mean *q_i,0_* (the initial habitat quality of site *x*) and the variance of σ^2^ = σ^2^_c_√(1−AC ^2^) with σ^2^_c_ = 0.15. We used q_x,0_ = 0.85 as a starting point, and we only considered highly predictable environments using AC = 0.8. The magnitude of environmental predictability has a low impact on local population dynamics over the time scale considered [10].

To improve the generalizability of our findings, we examined both a long- and a short-lived life cycle that may illustrate two typical contrasting cases at opposite ends of the slow–fast continuum [27]. We used demographic parameters of bird species available from the literature: the griffon vulture, *Gyps fulvus*, as a long-lived species [2], [28] and the barn swallow, *Hirundo rustica*, as a short-lived species [29] (see Table 1). These demographic rates have been previously used in general simulation models of contrasting life cycles [10], [24], [30], but parameters of other animal taxa, such as mammals, could have been employed instead. The demographic rates used were known to yield to a deterministically growing population (i.e., a growth rate higher than 1) under this regime of environmental quality without emigration or immigration [10] to ensure that local extinction was mainly the result of dispersal. As in [10], [30], we assumed a proportion of γ = 0.8 among mature individuals able to breed each year and a balanced primary sex ratio of 1∶1. When the breeding carrying capacity was reached, the surplus of potential breeders did not reproduce and were considered as floaters. Demographic parameters were independent of population size, and population regulation was designed based on the saturation of breeding sites [31]. Reproductive success was a function of habitat quality defined as f_x,t_ = f×q_x,t,_, with f representing mean fecundity (Table 1). Because of the critical role of stochastic processes in the persistence of small populations [14], demographic stochasticity was modeled using (1) a binomial function for survival events (mean s_a_ or s_j_), the primary sex-ratio (mean 0.5) and the reproduction in the long-lived species (mean f_x,t_) and (2) a Poisson function for the reproduction of the short-lived species (mean f_x,t_). Release costs have been often reported to reduce demographic parameters at different times after translocation in relation to the time spent in captivity [30], [32], [33]. Consequently, we accounted for the release cost on survival cs during the first year after release and on fecundity cf throughout the lifespans of both life cycles (see Table 1 and [10] for details). Translocated individuals were able to reproduce within a year of their release, and release costs were not transmitted to wild-born offspring.

**Table 1:** Demographic parameters used to model translocations of the long-lived and the short-lived species.

	Life cycle	
Demographic parameter	Long-lived	Short-lived
Adult survival (s_a_)	0.987	0.45
Juvenile survival (s_j_)	0.858	0.3
Fecundity (f)	0.8	8
Adult survival with release cost^a^	0.74	0.34
Adult fecundity with release cost^a^	0.51	4.08
Age at first breeding (in year)	4	1

Demographic parameters and release costs are similar to [10].

a Survival and fecundity suffering from release costs were respectively calculated as s_a_.(1- cs) and as f.(1- cf), with cs = 0.25 and cf = 0.49.

### Dispersal and habitat selection decisions

We used the most common definition of dispersal, expressed as any movement between habitat patches [34], to indicate movements between breeding populations. At each time t, individuals may either remain within their current population x or move to the other population y to reproduce at t+1 according to the staying probability Sp_x,t_ (the probability of dispersal from x to y was defined as Dp_x-y,t_ = 1−Sp_x,t_; Fig. 1). Because habitat selection cues may differ at different living stages as a result of breeding success or experience [6], we differentiated successful from failed breeders. We largely relied on the “win stay, lose shift” paradigm to describe breeders' decision-making processes [35]. For successful breeders, Sp_x,t_ = 1, meaning that every successful breeder was faithful to its breeding population. We assumed that for successfully breeding individuals in a population, there was no advantage in moving and that the social information possessed by the other population was not available to these individuals when prospecting (see also [24]). In contrast, failed breeders at time t may have benefitted from dispersal and may have had time to prospect in the habitat of the other population. As a result, these failed breeders were considered to behave similarly to non-breeding floaters at time t. At the beginning of the breeding season t+1, failed breeders and non-breeding floaters at time t made up a pool of potential dispersers able to disperse according to Dp_x-y,t_. Immature individuals were assumed to be faithful to their natal population until they reached sexual maturity. Immature individuals at time t that became sexually mature and decided to breed at t+1 (i.e., first breeders) did not have previous breeding experience but may have had time to prospect at time t. As a result, these first breeders were assumed to select their breeding population in a manner similar to that of failed breeders and non-breeding floaters at time t. The stochastic realization of dispersal events enabled us to represent inter-individual variations in the costs and benefits of dispersal [34]. Dispersal events were determined through a binomial function of the mean staying probability Sp_x,t_, defined as follows:
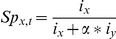
where i_x_ is the attractiveness of the current breeding population x according to the information criterion i defined by the behavioral rule considered (see below). Similarly, i_y_ is the attractiveness of the other population y, and α is the degree of connectivity between populations. The degree of connectivity α (ranging from 0 to 1) allowed for the weighting of the attractiveness of the neighboring population. Connectivity mimicked the effects of spatial barriers, neophobia, corridor design and distance on the perception of habitat cues and the resulting reduction in migration rates (however, see [12]). When α = 1, the two populations were closely connected and played similar roles in dispersal decisions. In contrast, when α = 0, populations were isolated, and their dynamics were independent.

We investigated four behavioral strategies by modeling i_x_ according to the information criteria considered for the given dispersal strategy employed by the individuals in the source population x. First, we considered the null hypothesis of a random dispersal strategy (Random strategy, i_x_ = 0.5). Second, we considered an ideal free-habitat selection strategy [36] in which individuals were able to optimally perceive all components of environmental quality (Quality strategy, i_x_ = q_x,t_). Finally, we defined two rules in which dispersal decisions were influenced by social constraints depending either on the conspecific attraction hypothesis (Conspecific attraction strategy, i_x_ = nb_x,t_) or on the habitat copying hypothesis (Habitat copying strategy, i_x_ = rs_x,t_) with nb_x,t_ representing the number of breeders (i.e., number of breeding females) and rs_x,t_ the reproductive success rate at time t in population x. Reproductive success was calculated as the number of offspring divided by the number of breeders for both life cycles. We assumed that habitat selection by the breeding population at t+1 was based on the information available to this population after the reproduction pulse in year t. Importantly, released individuals were forced to remain and breed into the *translocated* population during the year after their release. We assumed that releases occurred immediately prior to the reproduction period, thereby restricting prospecting behaviors, and that translocation management techniques provided post-release resources to encourage released individuals to remain in the release area in the short term. This hypothesis is therefore conservative in its inferences of extinction probabilities based on behavioral dispersal decisions.

### Release strategies and social environments

We investigated the effects of (i) the released group size (10, 20 or 30 females), (ii) the timing of the release (a single release with all individuals released in the same year versus the release of a consistent number of individuals over 10 years), (iii) the variation in the proportion of breeders among released individuals during the year of release (γ, ranging from 0.1 to 0.8; afterward, released individuals behaved as wild individuals with γ = 0.8), and (iv) different social environments by considering the initial size of the *remnant population* (from 0 to 30 females).

Model outputs included the global and local extinction probabilities (for both the *translocated* and the *remnant* populations). Simulations resulted from a 50-year horizon of 1000 Monte Carlo trajectories. Local extinctions were determined as the last individual of a given population leaving the population or dying, and recolonizations were allowed. When a population reached breeding-site saturation, the surplus of individuals remained non-breeding floaters unless they were attracted to the other population. Extinction probabilities were recorded beginning with the last release event for the *translocated* population or from the first colonization event of the *remnant* population when its initial size was set to zero.

## Results

We found qualitatively similar results for the short- and long-lived life cycles, although the response patterns are more pronounced for the long-lived species. As a result, our findings are presented for long-lived species only (short-lived outcomes are presented in Fig. S1 and Fig. S2). Although local population extinctions did occur, extinctions of the metapopulation never occurred in any scenario.

### Social-based dispersal behavior in a highly connected network

**Figure 2:**
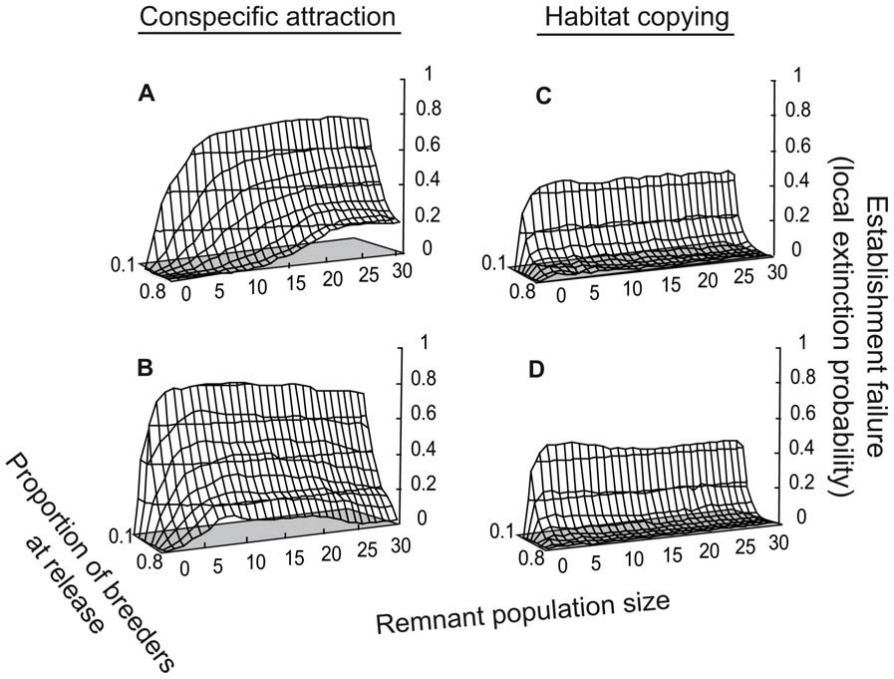
Long-lived establishment failure probabilities of *translocated* population for two social behaviours and two release methods. Sensitivity of establishment failure to different proportions of breeders at release and various *remnant* population sizes when *translocated* and *remnant* populations are totally connected (α = 1). Social behaviours: Conspecific attraction, based on the presence of conspecific breeders (A and B); Habitat copying, based on the reproductive success of conspecifics (C and D). Using a female life-cycle model, population size and release group size are expressed as a number of females. Release methods: single release of 20 females (A and C); sequential releases of 2 females/year during 10 years (B and D). Proportion of breeders at release acted among released individuals the year of release only, and was of 0.8 hereafter. Comparable results are presented for short-lived species in Fig. S1.

When the *translocated* and *remnant* populations are highly connected (α = 1), socially induced habitat selection may compromise the establishment of released individuals to the target area (Fig. 2). Conspecific attraction may lead to substantial risks of establishment failure (up to 80%) regardless of the release method considered. Under the conspecific attraction hypothesis, the relative efficiencies of the sequential and single-release strategies varied according to the size of the *remnant* population. For small *remnant* population sizes, the viability of the *translocated* population was minimized with sequential releases, whereas the reverse was true for large *remnant* population sizes (see Fig. 2A). Moreover, the extinction rate of the *translocated* population did not increase monotonically with the *remnant* population size under sequential releases but reached a maximum risk for intermediate *remnant* population sizes (Fig. 2B). This association between maximum extinction risk and intermediate population size never occurred with single releases (Fig. 2A). In contrast, the habitat copying decision-making strategy did not lead to strong differences in extinction probabilities among release methods, and the probabilities of establishment failure were lower in the habitat copying strategy than in the conspecific attraction strategy for both life cycles (reaching a maximum at 58%, Fig. 2D) even if the risk remained high. When there was initially no *remnant* population, the establishment of the released individuals in the release area always occurred with each of the four behavioral strategies (see, e.g., Fig. 2 and Fig. S1 for social behaviors), and the *translocated* population grew as it would in the absence of emigration. As expected for social behaviors, the initially empty suitable site was never colonized, which is in contrast to our findings for the Quality and Random strategies (not shown).

Overall, the proportion of breeders during the first year after release appeared to be the parameter to which establishment failure was the most sensitive for species selecting habitats based on social cues. For a given *remnant* population size, the risk of establishment failure can be more than 10 times higher for populations with low proportions of breeders than for those with high proportions (Fig. 2 and Fig. S1). Interestingly, when the two populations were closely connected, the Quality and Random strategies never led to the extinction of any population independently of the life cycle, the released methods, the *remnant* population size and the proportions of breeders among released individuals. Similarly, the *remnant* population never went extinct for either of the two social behavioral strategies (i.e., conspecific attraction and habitat copying).

### The sensitivity of local extinctions to connectivity, remnant populations and released group sizes

**Figure 3:**
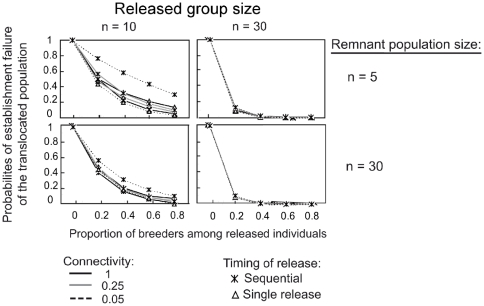
Long-lived species establishment failure probabilities of the *translocated* population for habitat copying behaviour. Sensitivity of establishment failure to the proportions of breeders at release and release methods for long-lived species, under different remnant population sizes and different levels of connectivity between populations. Similarly as in Figure 3, population size and release group size are expressed as a number of females. Timing of release: all females released in the same year (single release) or several releases of females during 10 years (sequential release). Connectivity affects dispersal to reach the neighbouring population and varies between 1 (full connectivity) and 0 (complete isolation). *Remnant* population sizes were the initial number of females in *remnant* population when simulations started. Extinction probabilities of the *remnant* populations were always equal to 0, and are then not represented. Establishment failures of the *translocated* population were local extinction probabilities.

**Figure 4:**
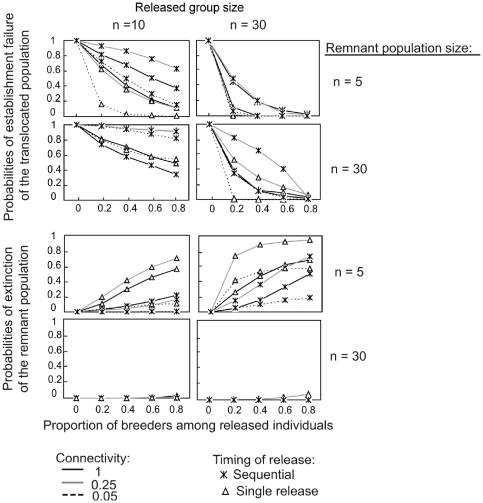
Long-lived species establishment failure and extinction probabilities of *translocated* and *remnant* populations for conspecific attraction. (Legend and simulations are similar to Figure 3.)

**Figure 5:**
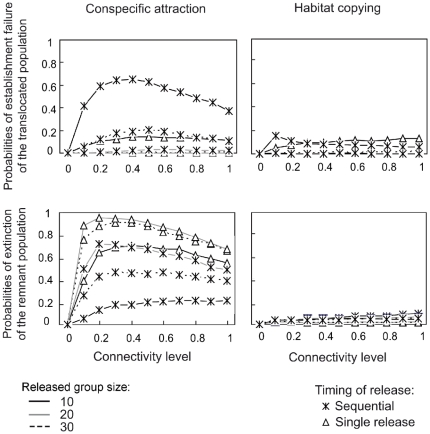
Long-lived species establishment failure and extinction probabilities for social behaviours given connectivity level. Sensitivity of establishment failure and extinction probabilities to the degree of connectivity between the translocated and the remnant populations and to different release methods. Legends and simulations similar to Figure 3 and 4, with a proportion of breeders at release of 0.8 and initial remnant population size of n = 5. *

Varying the connectivity between populations had little effect on the results presented above. Although connectivity increased local extinction risks, the response trends of the Habitat copying and Conspecific attraction strategies were mostly sensitive to the proportion of breeders among released individuals (Fig. 3 and Fig. 4, respectively). In contrast, both the Quality and Random strategies eliminated the extinction risk for both the *translocated* and the *remnant* populations in all scenarios tested. Under the Habitat copying strategy, the probabilities of establishment failure logically decreased with the increasing size of the released group, and there was no notable effect of the release method, the *remnant* population size, the degree of connectivity or the proportion of breeders in the release group (Fig. 3 and Fig. 5). The only exception was found with sequential releases of small groups in areas of low connectivity (n = 10 released females, α = 0.05; Fig. 3) for which the establishment of the *translocated* population may be compromised.

In contrast, under the Conspecific attraction strategy, the viabilities of the *translocated* and *remnant* populations showed complex responses to connectivity levels, partly because populations may go extinct in response to strong emigration or demographic stochasticity. Under a wide range of parameters investigated, an inverted u-shaped relationship linked connectivity to extinction probabilities for the two populations (Fig. 5). Local extinction probabilities reached their maximum at low to intermediate connectivity levels (0.1<α<0.4) and decreased or stabilized at higher levels. In addition, the effect of the level of connectivity on local extinction probabilities was strongly associated with the release method (Fig. 4 and Fig. 5). The single-release methods always improved the probability of the establishment of the *translocated* population at a given connectivity level but may simultaneously threaten the persistence of small *remnant* populations. This negative effect was primarily observed in long-lived species (see Fig. 4 and Fig. S2). Sequential releases have opposite trends to those found for single releases. The extinction probabilities of the *translocated* populations decreased as expected as the released group size increased. However, an increase in the group size was also associated with a higher risk of collapse for small *remnant* populations (Fig. 4).

## Discussion

### The vacuum effect

Integrating Conspecific attraction and Habitat copying strategies in decision-making criteria in stochastic dispersal rules strongly influenced individual distribution across populations, leading to either reduced establishment probabilities for the *translocated* population or increased extinction risk for the *remnant* population as compared with scenarios with random or habitat-quality-based selection behaviors. Conspecific attraction and Habitat copying, as both social and aggregative behaviors, may enhance one-way dispersal toward the most attractive conspecific population. This phenomenon, hereafter called the “vacuum effect”, was more important for Conspecific attraction than for Habitat copying. The Habitat copying strategy assumes that both the presence and reproductive performance of conspecifics influence dispersal decision. Because we assumed that temporally variable habitat quality affected reproductive performance, the effect of the attraction by conspecifics may be buffered by their relative reproductive performance and may cause substantial (re)colonization in both populations.

The influence of the vacuum effect was greater for the long-lived than for the short-lived species. Only non-breeding floaters and breeders who failed early were assumed to disperse based on likelihood that breeders decide to stay according to their experience in a particular environment [8]. As a result, effective dispersal rates (and the magnitude of the vacuum effect) depended on the relative proportions of experienced and inexperienced breeders in the population. As a result of slower population turn-over [14], long-lived populations are composed of a majority of older adults faithful to their breeding population after successful breeding and by relatively few dispersers compared to short-lived populations. Consequently, relatively higher numbers of dispersers in short-lived species enable a greater number of rescue effects before extinction.

The habitat-quality-based and random strategies did not affect the establishment modalities of the *translocated* population or the extinction probabilities of the *remnant* population provided that i) local environments had equal average quality and ii) there was no dispersal cost. On average, random strategies allocated half of each population to dispersers, preventing extinction by enhancing rescue effects. Because the average habitat qualities were equal, the habitat-quality-based strategy showed the same pattern. The habitat-quality-based strategy can be largely related to the ideal free distribution [36], and in our case, the habitat-quality-based strategy spread dispersers in a way that benefited the viability and establishment of both populations. When the population establishment is assumed to be complete, the Habitat copying and Quality strategies both aggregate animals in similar habitats and yield comparable fitness performances [10], [24]. Empirical observations of ideal habitat selection resulting from aggregations through social information have also been reported [37]. However, the vacuum effect highlights the fact that even though the Habitat copying and Quality strategies can lead to comparable distribution patterns and fitness performances in established populations, ignoring the causal mechanisms of animal behaviors can result in misleading projections of population persistence and extinction risk whenever population establishment remains uncertain.

Density-dependent dispersal is known to affect the probability of patch occupancy and extinction [11]. Dispersal induced by attraction to conspecifics has been identified as an inhibitor of patch colonization [12], [38] and yields a lower number of occupied sites in metapopulations [39]. In contrast, dispersal induced by conspecific cueing is known to enhance rescue effects and may lead to increased local population viability [11], [12]. In the present study, we found that the vacuum effect may either enhance or inhibit the rescue effect by increasing either the establishment or the persistence of some populations at the expense of others. Although our findings corroborate those of previous studies, we emphasize the fact that the vacuum effect resulting from conspecific cueing may reduce the number of occupied sites in a metapopulation not only by preventing the colonization of empty patches but also by accelerating the extinction of some small populations.

The intensity of the vacuum effect was proportional to the population size and inversely related to the connectivity between populations. Colonization and rescue effects induced by social behaviors have been shown to be inhibited by low levels of connectivity (e.g., habitat fragmentation) [9], [12]. Here, we found a non-monotonic relationship between connectivity and local extinctions, revealing interesting considerations for metapopulation restoration. The highest extinction probabilities occurred at intermediate levels of connectivity. At low levels of connectivity, local populations benefited from low emigration, whereas at high levels of connectivity, both populations inversely benefited from high immigration in compensation for the loss of emigrant individuals. At intermediate connectivity, however, the imbalance between emigration and immigration was at its peak. For small populations, the intermediate connectivity reduced the compensatory rescue mechanism before extinction. Our results corroborate previous findings by showing that dispersal in small populations can be disadvantageous when emigration exceeds immigration, making these populations more prone to dropping below a critical threshold size [40]. Importantly, we only focused on positive-density-dependent dispersal, whereas negative-density-dependent processes may also occur. Negative-density-dependent dispersal from large to small populations can enhance rescue effects and reduce the risk of extinction. Translocation can artificially play a similar role by countering the negative effect of emigration in small populations. A recent study has demonstrated that artificial increases in the number of groups in populations of the African wild dog (*Lycaon pictus*) can both improve the probability of suitable mating events and the production of successful dispersers, enabling the formation of new groups [5].

### The vacuum effect: another form of the behaviorally mediated Allee effect

Our simulations showed that local populations may go extinct and fail to establish as a result of the high emigration rates caused by social attraction. Several translocation failures resulting from post-release dispersal have been reported (see the review in [41]), and social attraction has been identified as the proximal cause in some cases. One empirical example of this phenomenon is the failure of Caribou (*Rangifer tarandus*) reintroduction programs in eastern North America because all of the animals joined nearby herds [42]. Additionally, repeated releases of 50 griffon vultures (*Gyps fulvus*) in 5 consecutive years in Navacelles (southern France) failed to establish a population because the released individuals joined a colony settled 50 km away that included at least 78 breeders at the beginning of the Navacelles project [3]. In a similar result, attempts to reinforce the Mediterranean metapopulation of the Audouin's gull (*Larus audouinii*) failed because the released birds preferred to emigrate to other breeding patches [4].

Because small populations are less attractive than large ones for behaviors based on social attraction, the local and negative effects of the vacuum effect increase as the local population size decreases. Behaviorally mediated Allee effects have been primarily described as preferences for poor habitat quality at low population density [22]. The behaviorally mediated Allee effect proposed by [22] and the vacuum effect both imply an increase in extinction risk resulting from the spatial distribution of individuals at a low population size. However, these effects differ in their causal mechanisms. The vacuum effect does not directly reduce any component of individual fitness and, therefore, does not represent a component Allee effect [15]. Rather, the vacuum effect may decrease the attractiveness of a site as a population becomes small and, as a result, directly affect the population at a demographic level (i.e., the vacuum effect acts as a demographic Allee effect). The vacuum effect extends the principle of the Allee effect to metapopulation systems when populations are defined as relatively small and even when no cost is associated with dispersal. We did not integrate the costs associated with movements (see [9], [34]). Our primary goal was to separate the costs associated with movements from the demographic consequences of the dispersal itself to highlight the impact of the vacuum effect. Moreover, integrating dispersal costs would have increased both local and global extinction probabilities, but costs are not expected to change our qualitative results if they occur independently of the decision to disperse. In addition, the vacuum effect may reciprocally increase the establishment and persistence probabilities of populations that attract the highest numbers of migrants. The number of connected populations, population size and asynchrony are the key elements of metapopulation viability [43]. Therefore, the vacuum effect may strongly affect the viability of a metapopulation system by modifying the number of populations involved in the networks and their connectivity through population extinction or colonization processes.

### Implications for management and translocation modeling

Release methods, with respect to release group size or release timing, can strongly impact the intensity of the vacuum effect. A single release event of a large group size enhanced immigration into the *translocated* population and endangered the viability of the *remnant* population, especially when the remnant population was small. Conversely, when few individuals were released sequentially, low levels of social information were produced, and the *translocated* population failed to retain individuals. This finding corroborates empirical observations in which repeated releases of large numbers of individuals were generally required to successfully establish a translocated population [1], [44]. Furthermore, released groups initially composed of a small proportion of breeders fail in 50 to 80% of cases in establishing individuals in a release site that is in the vicinity of a remnant population (Fig. 2).

Whenever translocations are intended to restore a connected population network at particular sites, such as in some reintroductions, the consequences of the vacuum effect should be assessed prior to release for both the translocated and the remnant population(s) and should account for the specificity of the species' behavior, the connectivity of the populations, and the environmental or conspecific context. Neglecting these effects may lead to the unexpected extinction of local populations and the subsequent failure to restore the metapopulation structure [5] and reduce the benefits of asynchrony between local population dynamics in species facing environmental stochasticity, habitat loss or fragmentation [45], [46]. Empirically, post-release movements have been reported to be affected by conspecific environments and to constrain the establishment of translocated populations (e.g., empirical descriptions of vacuum-like effects in which emigration is positively associated with the size of the closest established population [3], cases of population supplementation in which the fidelity to a release site is increased by the presence of conspecifics [47], and, inversely, observations of a density-dependent repulsive effect [48]).

According to our findings, species exhibiting aggregative socially based behaviors (e.g., colonial birds and social mammals) should be released in large groups of sexually mature animals with an imprinting period adequate to familiarize them with the proximal environment and to maximize local establishment [33], particularly in cases in which a large population exists close to the release area. However, the vacuum effect is not a factor for species in which social cues or conspecific density play a small role in habitat selection (e.g., reptiles and amphibians) or for which resource availability is a major determinant of translocation success (e.g., territorial species). Although translocation can represent a valuable tool for species conservation, from reintroductions to assisted colonizations [49], our results demonstrate that the conspecific vacuum effect can result in local restoration failure or cause collateral damage to existing populations [50]. In addition to estimating release-site quality, release management strategies should include a preliminary assessment of the risk of establishment failure to maximize the benefit to the conservation of the released species while minimizing collateral effects (e.g., local extinctions of indigenous species or remnant populations and dispersal-related conflicts with humans [51]). Given the limited availability of logistical and financial support, redefining the initial objectives and spatial scales of translocations may be of substantial benefit in decision-making and proactive adaptive management strategies.

## Supporting Information

Figure S1 **Short-lived establishment failure probabilities of** ***translocated*** **population for two social behaviours and two release methods.** (Legend and simulations are similar to Figure 2 with respect to in demographic parameters according the life-cycle).
(TIF)

Figure S2 **Short-lived species establishment failure and extinction probabilities of** ***translocated*** **and** ***remnant*** **populations for conspecific attraction.** (Legend and simulations are similar to Figure 3 with respect to differences in demographic parameters according the life-cycle).
(TIF)
