# Supplementary figures and images for: Post-Release Dispersal in Animal Translocations: Social Attraction and the “Vacuum Effect”
Source: PLoS One. 2011 Dec 14;6(12):e27453. doi: 10.1371/journal.pone.0027453 (PMC3237406; doi:10.1371/journal.pone.0027453)

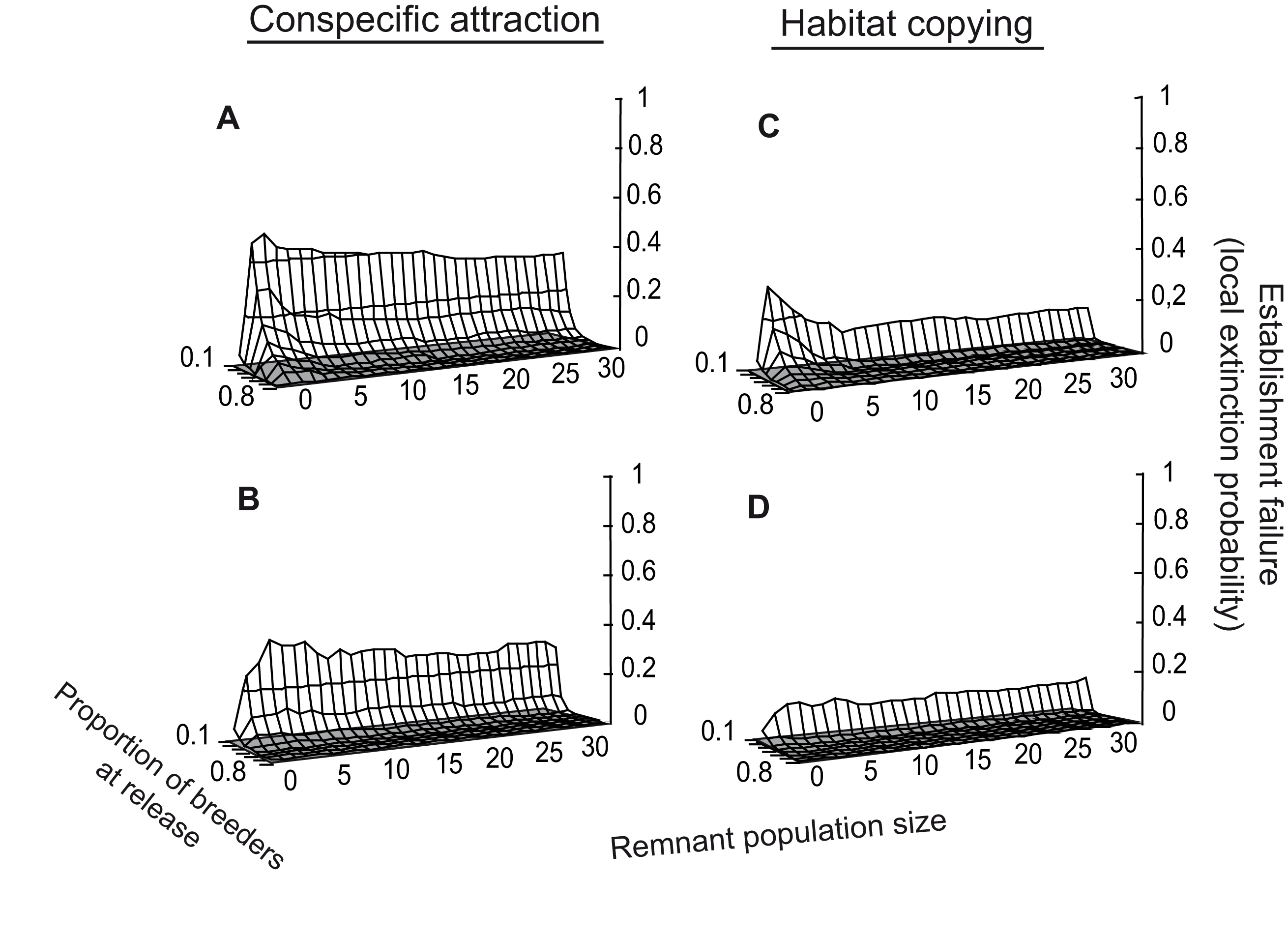

Supplement: Figure S1 — Short-lived establishment failure probabilities of translocated population for two social behaviours and two release methods. (Legend and simulations are similar to Figure 2 with respect to in demographic parameters according the life-cycle). (TIF) [file pone.0027453.s001.tif]

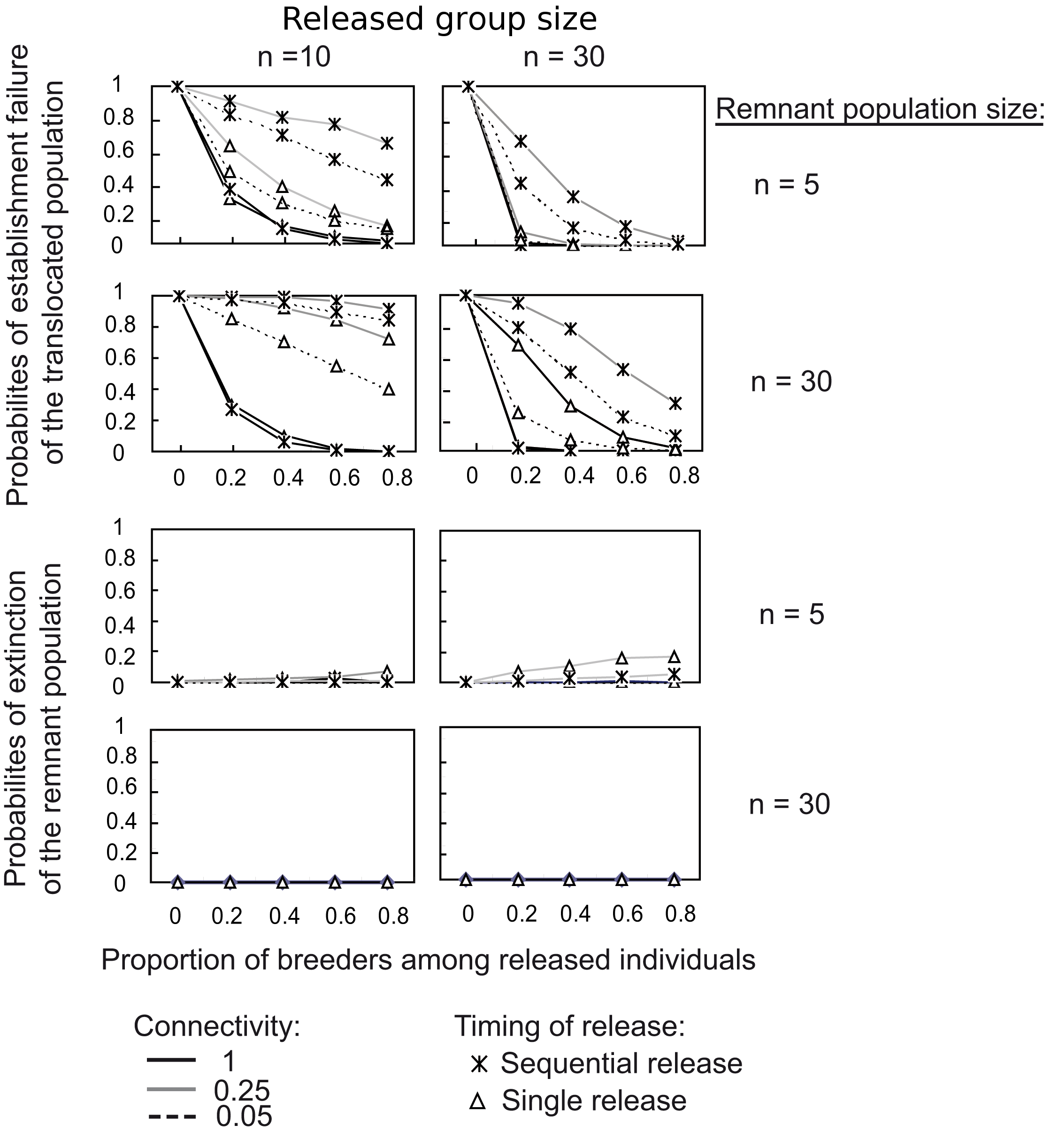

Supplement: Figure S2 — Short-lived species establishment failure and extinction probabilities of translocated and remnant populations for conspecific attraction. (Legend and simulations are similar to Figure 3 with respect to differences in demographic parameters according the life-cycle). (TIF) [file pone.0027453.s002.tif]
